# *Real-time* assessment of HER2 status in circulating tumor cells of breast cancer patients: Methods of detection and clinical implications

**DOI:** 10.1016/j.jlb.2023.100117

**Published:** 2023-10-08

**Authors:** Eleonora Nicolò, Mara Serena Serafini, Laura Munoz-Arcos, Letizia Pontolillo, Elisabetta Molteni, Nadia Bayou, Eleni Andreopoulou, Giuseppe Curigliano, Carolina Reduzzi, Massimo Cristofanilli

**Affiliations:** a Division of New Drugs and Early Drug Development, European Institute of Oncology IRCCS, Milan, Italy; b Department of Oncology and Hematology-Oncology, University of Milan, Milan, Italy; c Department of Medicine, Division of Hematology-Oncology, Weill Cornell Medicine, New York, NY, USA; d Medical Oncology Department, Catholic University of Sacred Heart, Fondazione Policlinico Universitario Agostino Gemelli IRCCS, Rome, Italy; e Department of Medicine, University of Udine, Via Chiusaforte, Udine, Italy; f Human Genetics Laboratory (LR99ES10), Faculty of Medicine of Tunis, University of Tunis El Manar, Tunis, 2092, Tunisia

**Keywords:** Circulating tumor cells, Breast cancer, HER2, HER2-Low, Prognostic value, Predictive value

## Abstract

The human epidermal growth factor receptor 2 (HER2) plays a central role in breast cancer (BC). Therefore, it is critical to develop a method that can capture its spatial and temporal heterogeneity. Nowadays, therapeutic decisions for BC patients relies on evaluation of HER2 status from tissue biopsies using immunohistochemistry and *in situ* hybridization. Nevertheless, considering the technical and logistical challenges associated with tissue biopsies, there is an unmet need for a non-invasive and accurate approach to obtain *real-time* assessment of HER2 status. In this context, circulating biomarkers, particularly circulating tumor cells (CTCs), emerged as promising candidates. HER2 assessment on CTCs can be performed at genomic, transcriptomic, and protein levels on both bulk CTCs and at the single-cell resolution. However, the main limitation of the literature to date is the lack of a consistent definition of HER2-positive CTCs, which poses a major challenge for both, future research and clinical applications. Several studies revealed discordance in HER2 status between the primary tumor and corresponding CTCs. For instance, HER2-positive CTCs have been detected among patients with HER2-negative BC and *vice versa*. As a result, researchers have evaluated the prognostic and predictive value of HER2 status in CTCs, both in the early and metastatic settings, to increase the possibility of using anti-HER2 therapy also for these patients and to dissect mechanisms of treatment resistance. This review aims to provide an overview of the methods to determine HER2 status in CTCs and to summarize the evidence and future perspective on how CTCs-HER2 assessment can be integrated into the clinical management of BC patients.

## Highlights

• HER2 expression has a central role in breast oncology.
• CTCs may allow a non-invasive and real-time assessment of HER2 status.
• HER2 status can be discordant between tissue and CTCs.
• Actionability of HER2-expressing CTCs is still unclear.
• Standardization of HER2 assessment of CTCs is needed.

## Introduction

Human epidermal growth factor receptor 2 (HER2) is a transmembrane tyrosine kinase receptor, member of the epidermal growth factor receptor (EGFR) family, encoded by the *ERBB2* gene located on chromosome 17q21. The dimerization of HER2 with other HER protein, mainly HER3, leads to the activation of downstream signaling pathways that promote cell proliferation and migration, and inhibit apoptosis [1]. HER2 protein overexpression and/or *ERBB2* gene amplification occurs in up to 20% of breast cancers (BCs), known as HER2-positive BCs [2]. Due to the dysregulated HER2 signaling these tumors are characterized by an aggressive behavior in the absence of treatment. Nonetheless, the development of drugs targeting HER2 has significantly improved the prognosis of patients with HER2-positive BCs [3]. In recent years, the development of novel anti-HER2 agents, namely antibody-drug conjugates (ADCs), reversed the dichotomous classification of HER2 status with the introduction of the new entity of HER2-low BCs with therapeutic implications [4]. These tumors represent approximately half of all BCs and are defined by low level of HER2 expression [*i.e.*, immunohistochemical (IHC) score of 1+ or 2+] and no detectable *ERBB2* amplification. Even if HER2-low tumors are not addicted to the HER2 pathway for cancer cells proliferation, patients with this subtype of BC may benefit from the HER2-targeted delivery of chemotherapy payloads [5].

Nowadays, the assessment of HER2 status and the selection of patients eligible for anti-HER2 therapy rely on IHC and *in situ* hybridization (ISH) on the primary tumor tissue according to the American Society of Clinical Oncology and the College of American Pathologists (ASCO/CAP) guidelines [6]. When metastasis occurs, it is recommended to reassess the receptor status through a tissue biopsy of a metastatic site before starting a new line of therapy [7]. However, in clinical practice, because of the technical and logistical challenges of tissue biopsies, decisions are often based on the receptor status of the primary tumor. Unfortunately, during disease progression, the molecular status of the tumor may evolve and become discordant with the primary site. This biological heterogeneity can further increase as a result of the molecular pressure created by systemic treatment [8]. HER2 overexpression is rather stable during the course of the disease, with primary-metastasis discordance found in up to 20% of cases [9,10]. Notably, a much higher dynamism has been reported for HER2-low and HER2-negative BCs with a relevant portion of HER2-low tumors turning HER2-zero, and *vice versa*, either on residual disease after neoadjuvant therapy [11], or following tumor relapse [[12], [13], [14]]. These observations underscore the crucial importance of reevaluating HER2 status during a patient's disease progression as inaccuracy or disease heterogeneity could lead to inappropriate treatment selection. Nonetheless, longitudinal monitoring of HER2 status is limited by the impracticality of repeated sampling of the tumor tissue or by the presence of inaccessible metastatic sites. Furthermore, tissue biopsies are influenced by the co-existence of cancer cells subpopulation with different HER2 expression across a single tumor location or different metastatic sites that can lead to discrepant HER2 status results depending on the analyzed region [15].

In the era of anti-HER2 ADCs, it is paramount to better capture the spatial distribution and temporal evolution of HER2 expression and a non-invasive and accurate approach to obtain a *real-time* assessment of HER2 status is thus an unmet need. In this context, the role of liquid biopsy stands out: among tumor-derived circulating biomarkers, circulating tumor cells (CTCs) can provide additional information to tissue biopsies to map tumor heterogeneity and evolution, better reflecting the HER2 status of the disease.

In this review, we provide an overview of the opportunities and pitfalls of different approaches to assess HER2 status on CTCs. Moreover, we summarize the latest evidence on the clinical utility of HER2 expression evaluation on CTCs in BC patients. Finally, we discuss potential future applications and challenges of HER2-positive CTCs in clinical practice.

## Approaches for the assessment of HER2 status on CTCs

CTCs are tumor cells that detach from the primary tumor and migrate in the bloodstream. They offer an easily accessible source of tumor-derived material in BC patients [16]. Because of CTCs' extreme rarity compared to other circulating cells, many systems have been developed to detect and isolate them. The enrichment and identification of CTCs rely on differences in size, density, electrical proprieties, molecular markers, and deformability compared to blood cells [17]. Most technologies for enrichment and identification also allow for CTC characterization at the protein, RNA or DNA level, depending on the methodology's characteristics. For example, the CellSearch® system [the first Food and Drug Administration (FDA)-cleared system for CTC detection and enumeration], uses an anti-epithelial cell adhesion molecule (EpCAM) antibody-bearing ferrofluid to enrich CTCs from peripheral blood samples [18,19]. After CTCs enrichment, immunophenotyping is performed through staining with fluorescently labeled antibodies against cytokeratins (CK), leukocyte marker (CD45), and 4′,6-diamidino-2-phenylindole (DAPI). CTCs are thus defined as nucleated cells (DAPI-positive), expressing CK but lacking CD45 expression, and can be further characterized through immunofluorescence (IF) staining for one additional marker (*e.g.*, HER2). However, the CellSearch® system precludes to perform RNA analysis, since cells are fixed. On the other hand, Parsortix®, the second FDA-cleared platform for CTC enrichment, utilizes an antigen-independent size-based microfluidics technology and, because it processes live cells, it allows for a variety of downstream applications/characterizations [20]. Nevertheless, other methods for CTC enrichment and assessment have been developed. Among them, the AdnaTest *BreastCancer*™ (AdnaGen AG, Germany) has been widely used in breast cancer CTCs studies [21]. The AdnaTest system™ performs CTC isolation, through an antibody mix linked to magnetic particles. The enriched cells are then lysed, and mRNA is extracted, transcribed into complementary DNA (cDNA) and multiplex polymerase chain reaction (PCR) is carried out for analyzing the expression of few and specific BC-associated genes. Even though it is an easy to perform technology, CTCs are collected and analyzed in bulk, consequently not allowing a single-cell resolution.

**Fig. 1:**
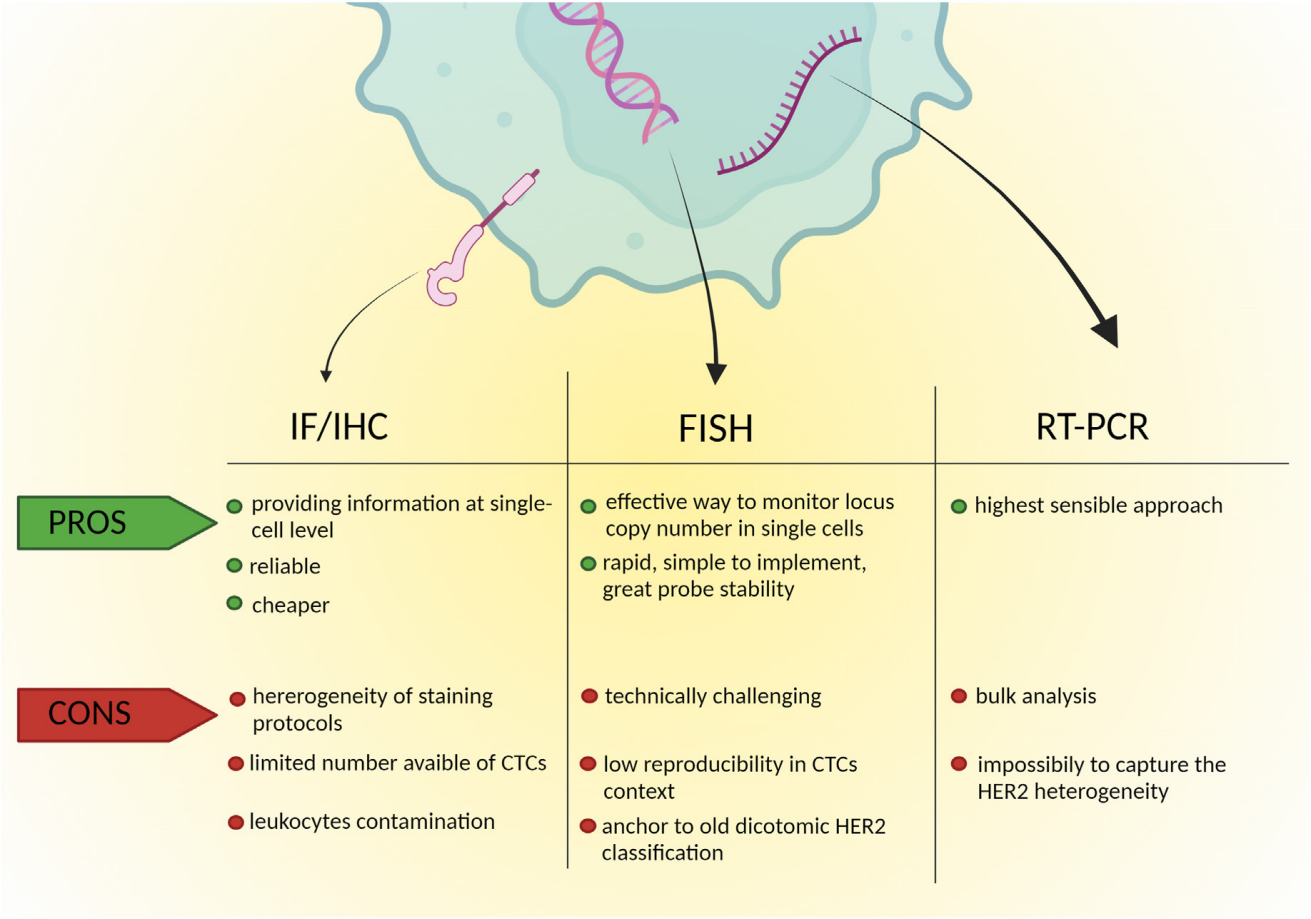
**Pros and cons of different methods for assessing HER2 status on CTCs.** Abbreviations: HER2: human epidermal growth factor receptor 2; CTCs: circulating tumor cells; IF: immunofluorescence; IHC: immunohistochemistry; FISH: fluorescence *in situ* hybridization; RT-PCR: reverse transcription polymerase chain reaction.

While the separation and enumeration of CTCs has been validated for use in clinical practice [19], the molecular characterization of CTCs (including the HER2 status assessment) is still not well established, even though it represents a unique opportunity for a comprehensive characterization of the tumor in *real-time*, which cannot be achieved with any other liquid biopsy analytes. With regards to HER2 assessment, CTCs can provide a simultaneous evaluation of (i) the presence of the protein on the cell surface, (ii) the expression of *ERBB2* at mRNA level, and (iii) the amplification of the *ERBB2* gene. Herein, we describe the available methods for HER2 status assessment on CTCs, indicating their strength and limitations (Fig. 1). Importantly, we highlight that currently there is no standardized and widely accepted method for determining the HER2 status on CTCs.

### HER2 expression on the cell membrane

The detection of HER2 protein on CTCs’ cell surface can be achieved through immunofluorescence (IF) or immunohistochemistry (IHC). These techniques have the advantage of providing information at the single-cell level, while being reliable and less expensive than other more sophisticated approaches (such as single-cell next generation sequencing). On the other hand, the heterogeneity of the staining protocols, the limited number of CTCs, and the presence of contaminating leukocytes in the samples make the use of these methods challenging. In a recent study, Chen et al. integrated their approach for detecting HER2 status starting from the purification of CTCs through the use LiquidBiopsy system (Cynvenio LLC, Westlake Village, CA) [22]. This system, identified as strength in their study, allows sufficient purity to perform a direct HER2 assessment using an anti-HER2 monoclonal antibody. The most widely used approach for the assessment of HER2 expression on CTCs through IF is the CellSearch® system, in which the CellSearch® tumor phenotyping reagent HER2/NEU assay (Menarini Silicon Biosystems) is incorporated into the staining cocktail. This technology has been employed in a broad range of studies, for both early and advanced BC [[23], [24], [25], [26], [27], [28], [29], [30], [31], [32], [33], [34], [35], [36]]. The CellSearch® system has an integrated fluorescence microscope and performs an automated scanning of all enriched cells, providing an image gallery of CTCs where HER2 fluorescence intensity for each CTC can be evaluated. Riethdorf and colleagues [23] were the first to categorize the intensity of the HER2 staining of CTCs analyzed with the CellSearch® into negative (0), weak (1+), moderate (2+), and strong (3+). This categorization was developed using BC cell lines with known HER2 gene amplification status confirmed by FISH analysis (MCF7, BT20, T47D, MDA-MB-453, SK-BR-3, and BT474 ​cell lines) and a high concordance between IF intensity and FISH was reported, supporting the accuracy of the IF intensity as a proxy of HER2 expression level. The HER2 scoring developed was adopted in subsequent studies, but it had to be performed by eye, and was therefore a qualitative and operator-dependent assessment.

To overcome this issue, an automated algorithm for image analysis was developed in 2017: ACCEPT (Automated CTC Classification Enumeration and PhenoTyping) is an open source software for the analysis of images acquired during the CellSearch processing, allowing, among other applications, for the quantification of specific markers expressed by CTCs, including HER2 [37]. Zeune et al. introduced the first application of ACCEPT to demonstrate the ability to extract relative expression of antigens expressed by CTCs. In particular, they used the HER2 mean intensity signal of three BC cell lines with a known HER2 IHC expression (SKBR-3 – 3+, MDA-MB 453–2+, MDA-MB 231–0 or 1+) for defining HER2 expression thresholds: i) HER2 negativity (0 or 1+) with a mean intensity of zero; ii) intermediate HER2-expression (2+) for a mean intensity between 0 and 100; iii) high HER2 expression (3+), with a mean intensity above 100 [37].

The results were further validated with flow cytometry, using BD Quantibrite™ Beads PE Fluorescence Quantitation Kit (BD Biosciences, San Jose, CA, USA), showing that the quantification of HER2 mean intensity using ACCEPT was a valid measure for HER2 expression. Successively, the authors compared the automatic score of ACCEPT with manual score performed by three clinical sites and observed a good agreement between the automated and manual scores. However, the manual score showed higher average HER2-positive cells than automated count, especially when clinical site 3 was considered (coefficient r = 0.82 and 0.98 for sites 2 and 3, respectively). It is worth mentioning that, in addition to the Marker Characterization tool (ACCEPT toolbox) allowing reproducible quantification of HER2 status on manually identified CTCs, it is also possible to apply the fully automated approach HER2 gates. Thus, it allows to obtain unified and objective scoring results, essential for the use in the context of multicenter studies.

A subsequent study compared the ACCEPT HER2 categories with gene expression and amplification using cell lines and blood samples from metastatic BC (MBC) patients [38]. Indeed, while previous studies demonstrated an association between HER2-positive CTCs based on visual scoring and HER2 gene amplification [39], the values of moderate HER2 expression needed to be explored. Brouwer and colleagues found that while HER2-high-expressing tumor cells demonstrated *ERBB2* amplification, CTCs with negative or intermediate HER2 expression were copy-number neutral [38]. Moreover, high relative *ERBB2* expression was detected in samples containing at least one HER2-high expressing CTCs.

Of note, these studies, and consequently their thresholds, were performed a few years ago, when BC was traditionally dichotomized into HER2-positive and -negative. Nowadays, with the introduction of the HER2-low BC “subtype”, much more attention should be dedicated also to the analysis of CTCs expressing low levels of HER2. Therefore, new studies should be conducted, aiming at better discriminating the different levels of HER2 expression. One recent study used the DEPArray^TM^ platform to establish thresholds able to discriminate also HER2 1+ CTCs [40].

Nowadays, considering the fast growth in the artificial intelligence (AI) and deep learning (DL) field, better tools/algorithms for images analysis might be developed.

Based on DL approaches, Jaber et al. established an algorithm based on PAM50 intrinsic subtyping using only whole-slide images of hematoxylin and eosin stained of the primary tissue [41]. Moreover, an AI tools was recently developed by Xue et al. utilizing HER2 gene amplification status obtained by FISH signals in tumor tissue [42]. The development of these approaches in the context of BC is opening the way to translate soon the acquired expertise to CTCs biomarkers analysis, obtaining more sensitive and reproducible tools for a refined HER2 quantification on CTCs, overcoming the categorization of CTCs into 0, 1+, 2+, 3+, for a continuous score of HER2 expression.

Another issue with respect to HER2 quantification in CTCs is the sample heterogeneity. Each CTC can in fact express a different level of HER2 within the same blood sample, raising the question of which should be the proper cutoff to consider a sample “HER2-positive” for therapeutic planning. A standardized cutoff has not been established yet. In the literature, some studies considered a blood sample as HER2-positive when at least 50% of CTCs showed HER2 expression [24,43] and recommended for an optimal HER2 evaluation the presence of at least 10 CTCs [43]. On the other hand, other studies established a threshold of only one HER2-positive CTC, without a minimal number of CTCs evaluated [23,44]. The lack of a consistent and widely accepted cutoff creates challenges in interpreting HER2 status from CTC analysis and warrants further research for standardization and clinical applicability.

Finally, single-cell proteomic analysis might constitute a more comprehensive approach to evaluate HER2 protein levels in CTCs, contextualizing the molecular heterogeneity beyond the mere HER2 status. A few methods for performing single-cell proteomics are under development [45,46], but this field has just started to evolve and need further extensive validation.

### *ERBB2* mRNA expression

As previously described, AdnaTest *BreastCancer*™ is an experimental method that, after the immunomagnetic-capture of the cells and their lysis, is based on mRNA extraction. The mRNA, successively converted in cDNA, is utilized for detecting specific marker using RT-PCR. One of the markers included is HER2. Different BC-based studies utilized AdnaTest *BreastCancer*™. As an example, in the trial conducted by Fehm and colleagues in 2009, they compared the results of CellSearch® and the *AdnaTest BreastCancer*™ assays when analyzing blood samples from 245 MBC patients [21]. The criteria for determining HER2-positivity were different between the two methods. With CellSearch®, CTCs were considered HER2-positive if at least five CTCs were detected and at least one of them showed strong HER2 staining (3+). On the other hand, with *AdnaTest BreastCancer*™, CTCs were deemed HER2-positive when the HER2 transcript was detected utilizing 2100 Bioanalyzer (Agilent Technologies, Santa Clara, CA, USA). Overall, they obtained results of HER2 status agreed in 64% of the cases. Of note, a drawback of this method is that it evaluates bulk CTCs and determines the average HER2 expression of all tumor cells, so it cannot capture HER2 heterogeneity, which, in contrast, would require evaluation of individual cells.

Strati et al. performed a multiplex RT-PCR, including *ERBB2* mRNA, and compared the mRNA expression with the protein expression detected using CellSearch® [47]. They found a higher positivity rate of HER2-positive CTCs with RT-PCR as compared to the CellSearch® assessment. Aaltonen et al. analyzed by multiplex qPCR 38 genes associated with cancer and 9 reference genes [48]. The customized gene panel was developed by the group. They found that the expression of HER2 mRNA in CTCs did not reflect the clinical diagnosis of HER2 status, and continuous evolutionary changes of HER2 mRNA were observed also in different patients, including those with triple-negative breast cancer (TNBC). Nevertheless, even if RT-PCR is the highest sensible approach to evaluate the HER2 mRNA expression, other methods have been utilized in the last decades, even if in the literature data remains scant due mostly to their technical difficulty. In 2005, Smirnov et al. utilized microarray on CTCs obtained from BC patients [49]. Recently, CTCs were utilized by Lang et al. to feasibly perform RNA-seq, and specific gene signatures have been identified [50]. However, for the biological analysis of CTC, these comprehensive approaches should be more frequently considered; indeed, the generation of a global gene expression profile, leads not only to the identification of CTC-specific genes but also supports the dissection of their biological heterogeneity. The gained knowledge will be especially useful in the context of HER2-low expression, identified by routinary methodologies, but still not fully understood.

### DNA amplification of the *ERBB2* gene

Another possibility to evaluate HER2 status in CTCs is the detection of *ERBB2* DNA amplification, mainly based on the use of FISH protocol specialized for CTCs to detect and map tumor cells in leukocyte background.

In 2019 Brouwer et al. compared HER2 IF signal with DNA amplification, showing that most of CTCs did not show HER2 amplification [38].

Interestingly, Mishima et al. developed a method to evaluate HER2 amplification in CTCs based on a three-dimensional (3D) multi-color cell imaging in a cohort of patients with gastric cancer [51]. CTCs were simultaneously labeled with IF antibodies (pan-cytokeratin and CD45) and a FISH probe for the HER2 gene (Spectrum Orange) and chromosome 17 centromere (Spectrum Green) as per the manufacturer's instructions (kit order #30–608,377/R7; Abbott Laboratories Inc., Des Plaines, IL, USA). The samples were then screened along the Z-axis using a 3D confocal scanning microscope and subsequently underwent 3D reconstruction, enabling the distinction of FISH signals that overlapped each other in the Z-axis direction. The 3D images were generated and analyzed by Fluoview software version 4.0 (Olympus). HER2 status was considered positive for gene amplification if the HER2:CEP17 ratio was ≥2. Interestingly, HER2 was evaluated also by immunofluorescence using CellSearch®, and the 3D–IF–FISH method detected a higher number of cases with HER2-negative primary tumor, but HER2-positive CTCs compared to CellSearch®. The technical difficulty and the low reproducibility of FISH in CTCs context is mirrored by the scant data available in the literature. Moreover, the investigation of HER2 amplification is still anchored to the old dichotomic HER2 classification, not considering the phenotypic heterogeneity of HER2 expression in BC unrelated to genomic differences.

## Defining HER2 status: from the tissue to CTCs

**Fig. 2:**
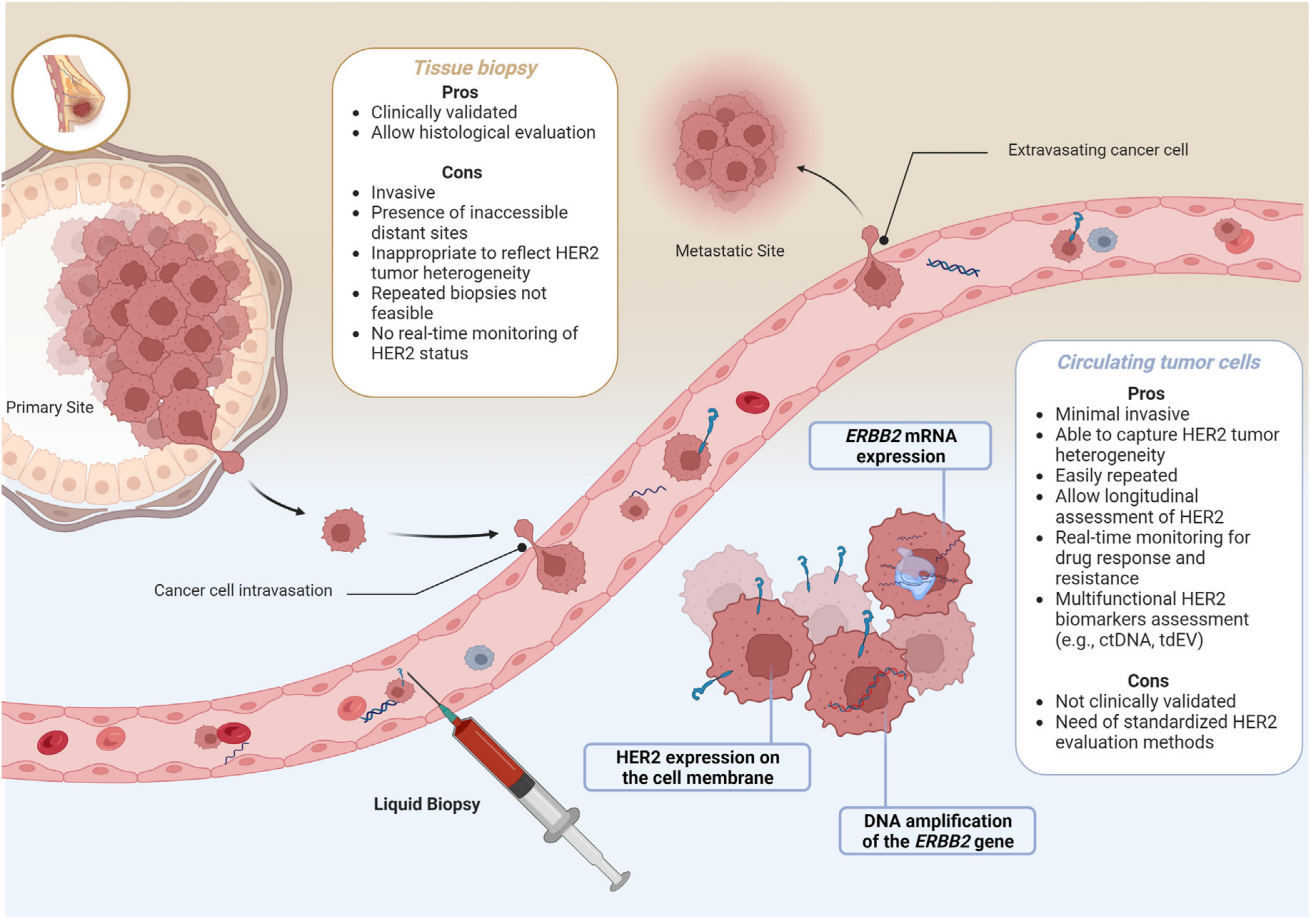
**HER2 assessment in Breast Cancer: from Tissue to Circulating Tumor Cells.** Abbreviations: HER2: human epidermal growth factor receptor 2; ctDNA: circulating tumor DNA; tdEV: tumor-derived extracellular vesicles.

HER2 status of the tumor directly influences the therapeutic strategy for patients with BC [7]. Nevertheless, tissue biopsies are unsuitable to capture spatial and temporal heterogeneity of HER2. The use of CTCs holds the promise of enabling a safe, repeated, and reliable assessment of HER2 status over time (Fig. 2). Thus, initial studies on HER2 expression on CTCs aimed at testing concordance with the tissue to evaluate the reliability of using CTCs for HER2 status definition (Table 1).

**Table 1:** Correlation of HER2 expression between tissue and CTCs

Author; year Ref.	Setting	CTCs enrichment method	CTCs detection rate (%)	HER2 assessment method	N of pts with HER2- positive CTCs (%)	Tissue evaluated for HER2 expression	N of pts with				Concordance ♦
							HER2 positive CTCs/HER 2 positive tumor	HER2 negative CTCs/HER 2 negative tumor	HER2 positive CTCs/HER 2 negative tumor	HER2 negative CTCs/HER 2 positive tumor	
Aktas et al., 2016 [55]	MBC	AdnaTest BreastCancer™	36/84 (43)	RT-PCR	18/36 (50)	Primary and Metastasis	Primary 8/14 Metastasis 8/14	Primary 12/20 Metastasis 14/22	Primary 8/20 Metastasis 8/22	Primary 6/14 Metastasis 4/14	Primary 20/34 (59) Metastasis 22/36 (61)
Chen et al., 2019 [22]	EBC and MBC	Microfluidic immunomagnetic (Liquid Biopsy system)	Not reported	Immunofluorescence	28/71	Primary	13/31	25/40	15/40	18/31	38/71 (54)
De Gregorio et al., 2017 [29]	HER2 negative MBC	CellSearch® system	711/1123 (63.3)	Immunofluorescence	134/711 (18.8)	Primary	Not applicable	577/711	134/711	Not applicable	577/711 (81)
Fehm et al., 2009 [21]	EBC	AdnaTest BreastCancer™	58/431 (13)	RT-PCR	22/58 (38)	Primary	Not reported	Not reported	Not reported	Not reported	53%
Georgoulias et al., 2012 [54]	HER2 negative EBC	Cytospin	148/378 (39)	Immunocytochemistry	51/57 (89)	Primary	Not applicable	6/57	51/57	Not applicable	6/57 (11)
Hayashi et al., 2012 [28]	MBC	CellSearch® system	31/52 (59.6)	Immunocytochemistry and FISH	8/31 (15.4)	Primary	5/9	19/22	3/22	4/9	24/31 (77)
Jaeager et al., 2017 [25]	HER2 positive EBC	CellSearch® system	258/642 (40.2)	Immunofluorescence	149/258 (57.8)	Primary	149/258	Not applicable	Not applicable	109/258	149/258 (58)
Krishnamurthy et al., 2013 [52]	EBC	Density gradient (Percoll) ​+ ​Microfluidic (OncoCEE)	26/95 (27)	FISH	6/88 (6.9)	Primary	1/9	74/79	5/79	8/9	75/88 (85)
Meng et al., 2004 [43]	MBC	Immunomagnetic	Not reported	Immunofluorescence and FISH	11/33	Primary	11/15	18/18	0/15	4/15	29/33 (88)
Munzone et al., 2010 [26]	MBC	CellSearch® system	57/76 (75)	Immunofluorescence	19/57	Primary	13/15	36/42	6/42	2/15	49/57 (86)
Pestrin et al., 2009 [24]	MBC	CellSearch® system	40/66 (61)	Immunofluorescence	15/40 (37)	Primary	7/12	20/28	8/28	5/12	27/40 (68)
Riethdorf et al., 2010 [23]	EBC	CellSearch® system	46/213 (21.6)∗	Immunofluorescence and FISH	81/37 (21.6)	Primary	3/11	21/26	5/26	8/11	24/37 (65)
Wallwiener et al., 2015 [27]	CTCs positive (≥5) MBC	CellSearch® system	107/107 (100)	Immunofluorescence	37/107	Primary and Metastasis	Primary 10/16 Metastasis 2/6	Primary 64/91 Metastasis 32/40	Primary 27/91 Metastasis 8/40	Primary 6/16 Metastasis 4/6	Primary 74/107 (69) Metastasis 34/46 (74)
Wülfing et al., 2006 [53]	EBC	Combined Buoyant gradient and immunomagnetic separation	27/35 (77)	Immunocytochemistry	17/35 (48.6)	Primary	2/3	12/24	12/24	1/3	14/27 (52)

Abbreviations: CTCs: circulating tumor cells; EBC: early breast cancer; FISH: Fluorescent *In Situ* Hybridization; MBC: Metastatic breast cancer; N: number; RT-PCR: Reverse transcription polymerase chain reaction.

♦Concordance: number of pts with concordant CTCs and tissue HER2 status/number of total.

∗: Before neoadjuvant treatment.

1: Considered as HER2 positive CTC only: “CTC HER2-strongly positive (3+) “

2: Result reported only for AdnaTest™.

3: Result reported only for baseline timepoint.

### HER2 expression on CTCs in early breast cancer

A variable discordance between CTCs and primary tumor HER2 status was observed in early-stage BC, irrespective of the different methods used across different studies. One of the first studies conducted by Fehm et al. using the *AdnaTest BreastCancer*™ in a large cohort of early BC (EBC) patients demonstrated a concordance rate of 53% between HER2 status of CTCs and primary tumor [21]. In a subsequent study by Krishnamurthy et al. a 15% of discordance between the primary tumor and CTC HER2 status was observed in EBC patients. In this study a microfluidic platform (OncoCEE™ microchannel technology) was used for enrichment of CTCs and HER2 status of CTCs was defined by FISH [52]. Wulfing et al. reported, in a cohort of stage I-III BC patients, a significant disagreement between HER2-positive CTCs detection, done using the combined buoyant density gradient and immunomagnetic separation procedure, and tissue's HER2 status [53]. Additionally, in a phase II trial evaluating early-stage BC patients with HER2-negative primary tumor, 89% of patients had HER2-expressing CTCs [54]. In this trial CK mRNA-positive CTCs were detected using RT-PCR and HER2 expression was evaluated by immunofluorescence. In a molecular analysis of the neoadjuvant GeparQuattro trial, an evaluation of the HER2 status on CTC and on corresponding primary tumors was performed: a discordance of 19.2% was observed in 26 HER2-negative patients while 5 of 11 (45.4%) HER2-positive patients had CTCs classified as HER2 0 or 1+ [23]. Also, a translational analysis of the phase III SUCCESS B trial reported a considerable discordance between the HER2 status of the primary tumor and CTCs in HER2-positive EBC patients before the start of adjuvant chemotherapy. Among the 258 CTC-positive (≥1 CTCs/30 mL) patients enrolled in this trial, 58% had at least one CTC with strong HER2 staining, 30% had moderate-to-weak HER2 staining, while 12% had exclusively HER2-negative CTCs [25].

### HER2 expression on CTCs in advanced breast cancer

One of the first studies addressing HER2 status on CTCs in advanced BC, using both immunofluorescence and FISH, was conducted by Meng and colleagues in 2004. After immunomagnetic enrichment for CTCs in a cohort of 31 BC patients, they observed a 97% concordance between tissue and CTC on HER2 status. Moreover, they found that 9/24 patients diagnosed with HER2-negative BC, acquired an *ERBB2* gene amplification in CTCs at disease progression [43]. In the study of Chen et al., 71 samples from patients with stage III-IV BC were characterized for HER2 expression in both primary tumor and CTCs analyzed through the LiquidBiopsy system (Cynvenio LLC, Westlake Village, CA); CTCs were defined as HER2-positive based on HER2 immunofluorescence intensity ≥3.5 times higher than CD45 intensity (100% sensitivity and 99.9% specificity on cell lines experiments). Among patients with HER2-positive BC (n = 31), 41.9% had ≥1 HER2-positive CTC as compared to 37.5% in patients with HER2-negative BC (n = 40). A higher number of HER2-positive CTCs (≥3) was found in 25.8% of HER2-positive BC, while in HER2-negative patients only one (2.5%) was found to have ≥3 HER2-positive CTCs. The authors stated that the frequency of HER2-positive CTCs was lower than the one reported in previous studies [22].

However, most of the studies evaluated the correspondence of the HER2 status between tissue and CTCs using the CellSearch® assay as enrichment method and the HER2 expression was manually scored. In a prospective longitudinal study of advanced BC patients, the concordance of HER2 status between the primary tumor and CTCs was 86% at baseline and 82% during treatment, with 18% of patients that acquired HER2 overexpression on CTCs after anti-HER2 treatment. The overall discordance rate was 18% [26]. Wallwiener et al. performed a retrospective analysis on 107 CTC-positive (≥5 CTCs/7.5 mL of blood) samples from MBC patients observing an overall HER2 expression concordance between CTCs and primary and metastatic tumor tissue of 69% and 74%, respectively [27]. A sample was defined as HER2-positive if at least one CTC showed moderate (2+) or strong (3+) staining according to criteria defined by Riethdorf et al. [23]. Results from a multicenter German study suggested how the concordance of the HER2 status between CTCs and both primary and metastatic tumor was influenced by the technique applied. Using the Cell Search® assay a non-significant concordance of 58% and 53% was reported when HER2 CTCs status was compared with primary and metastatic ones. On the other hand, utilizing AdnaTest™, the concordance rate of HER2 status CTCs was 59% (p = 0.262) and 67% (p = 0.04) with primary and metastatic tissue respectively [55]. Hayashi and colleagues evaluated a prospective cohort of MBC patients showing at baseline a discordance of 13.6% between HER2-negative primary tumors and HER2-positive CTCs, in contrast, 44.4% of patients with HER2-positive primary tumors had HER2-negative CTCs [28]. A similar discordance rate was reported also by Pestrin and colleagues: 29% of patients with HER2-negative primary tumors were positive for HER2 CTCs evaluation, whereas 42% of those with HER2-positive primary were negative on CTCs evaluation (k = 0.278) [24]. De Gregorio et al. characterized 1123 HER2-negative MBC patients for CTCs, defining the HER2 discordance with the tissue as the detection of at least one CTC with a strong HER2 immunocytochemical staining intensity (3+). Overall, 711 (63.3%) of patients had ≥1 CTC of whom 134 (18.8%) had at least one detectable HER2-positive CTC. Interestingly, discordance in HER2 phenotype was significantly associated with histological subtype [lobular vs ductal; odds ratio (OR) 2.67, p ​< ​0.001], hormone receptor (HR) status (positive vs negative; OR: 2.84, p ​= ​0.024) and CTC number (≥5 vs 1–4; OR: 7.64, p ​< ​0.001) [29].

Overall, these data highlight the need to define a cutoff for considering a sample as HER2-positive based on CTCs and to obtain an objective and reliable quantification of HER2 expression using automated algorithms for image analysis.

## Prognostic and predictive role of HER2 expression on CTCs

In patients with BC, the enumeration of CTCs using the FDA-approved CellSearch® system has demonstrated to be a strong independent prognostic factor in both the early and metastatic setting [19]. Despite this, to broaden the clinical utility of CTCs in guiding therapy decisions is relevant to explore the molecular features of CTCs, such as the HER2 expression - a well-established biomarker of prognosis and treatment response in BC (Table 2).

**Table 2:** Prognostic and predictive role of HER2 expression on CTCs

Author; year Ref.	Study design	Setting	CTCs enrichment method	HER2 assessment method	Prognostic value	Predictive value
Agelaki et al., 2015 [70]	Prospective	HER2 positive CTCs MBC	Gradient density centrifugation (using Ficoll)	Immunofluorescence	NA	No objective response in 22 pts treated with lapatinib
Apostolaki et al., 2007 [59]	Prospective	EBC	Gradient density centrifugation (using Ficoll)	RT-PCR	The incidence of clinical relapses was significantly higher in patients with detectable than nondetectable HER2 mRNA-positive CTCs (40% vs 19% p = 0.004) with a shorter DFS (p = 0.0058)	NA
Azim JR et al., 2013 [58]	Phase III trial	HER2 positive EBC	Modified Ficoll ​+ ​CellSearch® system	Immunofluorescence	NA	No pCR in the 3 HER2 positive CTCs pts
Beije et al., 2016 [32]	Retrospective	HER2 negative MBC	CellSearch® system	Immunofluorescence	Not significant prognostic value in the ET and chemotherapy cohorts for HER positive vs HER2 negative CTCs	NA
Georgoulias et al., 2012 [54]	Phase II trial	HER2 negative EBC	Cytospins	Immunocitochemistry	NA	DFS was significantly longer in the adjuvant Trastuzumab vs observation arm in pts with detectable CK19 mRNA-positive cells (p = 0.008)
Hayashi et al., 2012 [28]	Prospective	MBC	CellSearch® system	Immunocytochemistry and FISH	Negative PFS: p = 0.001 OS: p = 0.013	NA
Jacot et al., 2019 [71]	Phase II trial	HER2 negative MBC with HER2 positive CTCs	CellSearch® system	FISH	NA	1/11 pts treated with TDM-1 had PR. No difference in OS and PFS
Liu et al., 2013 [30]	Prospective	HER2 positive MBC	CellSearch® system	Immunofluorescence	Among pts receiving anti-HER2 therapy, HER2 positive CTCS were correlated with a better PFS (p = 0.002)	Among pts with HER2 positive CTCs: PFS longer for who received anti-HER2 therapy (p = 0.001)
Muller et al., 2021 [34]	Prospective	HER2 negative MBC	CellSearch® system	Immunohistochemistry	Pts with ≥1 CTCS with strong HER2 staining had shorter OS than those with CTCs with negative-to-moderate staining only (p = 0.013)	NA
Munzone et al., 2010 [26]	Prospective	MBC	CellSearch® system	Immunofluorescence	Negative Pts with HER2-positive CTCs had shorter PFS (p = 0.0019) and OS (p = 0.013)	NA
Parson et al., 2021 [36]	Phase II trial	HER2 negative MBC with HER2 positive CTCs	CellSearch® system	Immunochemistry and FISH	No difference in OS between pts with HER2 positive and HER2 negative CTCs (p = 0.56)	ORR 5% in pts treated with Trastuzumab-Vinorelbine
Pestrin et al., 2012 [35]	Phase II trial	HER2 negative MBC with HER2 positive CTCs	CellSearch® system	Immunofluorescence and or FISH	NA	No objective response in 7 pts treated with lapatinib
Riethdorf et al., 2010 [23]	Phase III trial	EBC	CellSearch® system	Immunofluorescence and FISH	NA	HER2 positive primary tumor and HER2 positive CTCs higher pCR (not statistically significant)
Wang et al., 2020 [33]	Prospective	HER2 negative MBC	CellSearch® system	Immunofluorescence	PFS shorter for HER2 positive CTCs vs HER2 negative CTCs (p = 0.013)	Among patients with high-risk disease and HER2- positive CTCs who underwent anti-HER2 therapy has longer PFS (p = 0.035)
Wulfing et al., 2006 [53]	Prospective	EBC	Combined Buoyant gradient and immunomagnetic separation	Immunocytochemistry	Significant correlation between the presence and frequency of HER2 positive CTCs and both a decreased DFS (p = 0.007) and OS (p = 0.024)	NA
Zhang et al., 2016 [31]	Prospective	HER2 positive MBC	CellSearch® system	Immunofluorescence	PFS longer for HER2 positive CTCs pts (p = 0.0011)	Among pts underwent anti-HER2 therapy longer PFS for HER2 positive CTCs vs HER2 negative CTCs: p < 0.001

Abbreviations: CTCs: circulating tumor cells; EBC: early breast cancer; FISH: Fluorescent *In Situ* Hybridization; MBC: metastatic breast cancer; pCR: pathological complete response, PFS: progression free survival; pts: patients; PR: partial response; NA: not assessed, ORR: objective response rate; OS: overall survival; RT-PCR: reverse transcription polymerase chain reaction.

### HER2-positive CTCs in early breast cancer

The majority of BCs are diagnosed in the early stages and undergo curative treatment. Despite remarkable advancement in the management of EBC patients, roughly 30% will develop distant recurrence, which accounts for 90% of BC-related death [56,57].

Some studies have evaluated the prognostic value of the detection of HER2-positive CTCs in the early setting. In the GeparQuattro trial, patients with HER2-positive tumors and HER2-positive CTCs at baseline had a tendency to a higher pathological complete response (pCR) rate after neoadjuvant therapy plus trastuzumab compared with patients with HER2-negative CTCs [23]. A sub-study from the NeoALTTO trial found that none of the three patients with detectable HER2-positive CTC at completion of neoadjuvant treatment achieved pCR [58]. Moreover, a reduced disease-free survival has been reported for EBC patients with HER2-positive CTC defined by IHC [53] or HER2 mRNA-positive CTCs [59]. However, due to the limited number of patients evaluated in these studies no definitive conclusion on the impact on outcome of HER2-positive CTCs in EBC can be derived.

Also, some unexpected failures of systemic treatments of EBC patients may be explained by intratumor heterogeneity of HER2 expression [60,61] that can be addressed through CTCs. Primary tumors with heterogenous HER2 expression may indeed shed CTCs with different HER2 status leading to the finding of HER2-positive CTCs in HER2-negative BC patients and *vice versa*. Therefore, CTCs may give a more precise picture of the HER2 status of a patient and inform on the more appropriate (neo)adjuvant treatment. So far, no data is available on the effect of personalized treatment based on the HER2 phenotype of CTCs in the early setting.

In an interesting preliminary study, it was observed that patients with HER2-negative EBC and detectable CTCs (cellular residual disease) treated with trastuzumab after adjuvant chemotherapy had reduced risk of disease recurrence as compared to observation [54]. In this study, the administration of trastuzumab resulted in a significantly decreased number of patients with detectable CTCs which could be attributed to the fact that almost 90% of the evaluated patients had HER2-expressing CTCs. An analysis of patients with HER2-negative EBC found that approximately 75% of CTC-positive patients had CTCs expressing HER2 and despite the mean CTC count decreased after 10 years, the population of HER2-positive CTCs increased during the follow-up [62]. Although the clinical meaning of HER2-positive CTCs in HER2-negative EBC is unclear, this finding may suggest that targeting HER2-positive CTCs may have implications for the prevention of late relapses.

Similarly, among HER2-positive BC patients treated with adjuvant trastuzumab, the persistence of HER2-negative or weakly positive CTCs may suggest a therapy-induced selection of HER2-negative tumor cells while the persistence of HER2-overexpressing CTCs could indicate a resistance to this type of treatment [23].

A proof-of-concept study in early TNBC patients showed that CTCs detected after neoadjuvant treatment shared more genomic alterations with residual disease than primary tumor, suggesting that these cells represent resistant clones [63]. Thus, evaluation of therapeutic targets such as HER2 on these cells might enable an individualized and optimized treatment to prevent relapse increasing the cure rate of patients with BC.

A limitation of this approach is the low CTC detection rate (20–40%) in the early setting compared to the metastatic setting. In several blood samples from EBC patients, only one CTC is detected which makes the evaluation of HER2 status even more challenging [23,64,65]. Even if usually the amount of blood used to count CTCs is 7.5 mL, reaching a higher volume (*e.g.*, 30 mL as in the SUCCESS B trial) may increase the detection rate and sensitivity.

### HER2-positive CTCs in metastatic breast cancer

Using the CellSearch® system approximately half of patients with MBC have ≥5 CTCs/7.5 mL, the validated cutoff to differentiate patients with favorable and unfavorable prognosis in the metastatic setting [19]. Considering the strong prognostic and predictive value of HER2 status in MBC different studies have explored the impact of having CTC expressing HER2.

#### Prognostic value of HER2 expression on CTCs in MBC

Regarding the prognostic role, initial studies reported a shorter progression-free survival (PFS) for those patients with HER2-positive CTC at baseline and 3–4 weeks after treatment initiation [26,28]. However, these studies included a mixed BC population concerning HER2 status on tissue biopsy, while the biological significance of HER2-positive CTCs might be different based on the BC subtype.

In HER2-positive BC patients, the presence of HER2-positive CTCs before starting an anti-HER2 treatment was associated with longer PFS [30,31].

In patients with HER2-negative MBC receiving either endocrine therapy (n = 72) or chemotherapy (n = 82), a similar PFS was reported regardless of the presence of HER2-positive CTCs [32]. Differently, Wang and colleagues found that patients with HER2-negative MBC treated with standard-of-care therapy (n = 105) had an increased risk of disease progression (HR: 2.16, p = 0.01) and a shorter overall survival (OS) when ≥2 HER2-positive CTCs were detected [33]. This study also suggested for these patients with ≥2 HER2-positive CTCs a benefit from the administration of anti-HER2 targeted therapies (HR: 0.30, 95% CI 0.10–0.92, p = 0.035), while no PFS improvement was observed for low-risk patients (<2 HER2-positive CTCs). Moreover, longitudinal analysis revealed as high-risk patients who had clearance of HER2-positive CTCs exhibited a significantly longer survival compared to those with persistence of ≥2 HER2-positive CTCs. A similar prognostic and predictive role of HER2-positive CTCs has been reported by the DETECT study group. At least one strong HER2-positive CTC was found in 15% of patients with HER2-negative MBC screened for participation in the DETECT III and IV clinical trials (n = 1933) and was associated with worse OS (HR: 1.36, p = 0.013) in the univariate but not in multivariate analysis [34]. Differently, the proportion of HER2-positive CTCs among all CTCs was not associated with clinical outcome. Interestingly, patients with hormone receptor-positive tumor were more prone to present strong-stained HER2 CTCs.

Moreover, with the introduction of the HER2-low paradigm interest has been raised in those CTCs with low HER2 expression and a negative prognostic value has been suggested [66]. In a retrospective analysis of MBC patients, a significantly longer PFS was reported for patients with >75% of CTCs having moderate or strong staining (*i.e.*, 2+ or 3+) as compared to those with >25% of CTCs with weak or negative HER2 (*i.e.*, 1+ or 0). Also, the presence of HER2-low CTCs (1+) has been associated with a distinct and more aggressive pattern of metastatic spread [67]. These findings suggest a peculiar biological meaning of CTC-HER2 1+, thus exploring their nature could allow us to better understand the role of these cells in the natural evolution of the MBC. D'Amico et al. using the DEPArray™ (Menarini Silicon Biosystems, S.p.A.) developed a pipeline to distinguish and collect HER2-low CTCs that would allow to individually select these cells and perform downstream molecular analysis [68].

#### HER2 expression on CTCs and response to anti-HER2 therapy in MBC

In the phase III DETECT III trial, 105 HER2-negative MBC patients with concomitant detection of HER2-positive CTCs were randomized to receive standard-of-care therapy alone or combined with lapatinib. An initial report from this study showed a tendency to a better PFS (HR: 0.69, p = 0.14) and a significantly improved OS confirmed by the multivariable analysis (HR: 0.55, p = 0.016) in the lapatinib arm compared to patients in the standard arm [69]. The rate of clearance of CTCs at the end of the study treatment, which was the primary endpoint of this trial, did not differ significantly between the two arms.

Nonetheless, the predictive role of HER2-positive CTCs is still debated considering other trials that reported a lack or minimal benefit of the administration of anti-HER2 agents in this patient population. In two different studies, no objective responses were observed with the administration of lapatinib in patients with HER2-negative MBC and HER2-positive CTCs [35,70]. A significant decrease in HER2-positive CTCs number with lapatinib treatment was observed only among patients with disease stabilization in the study of Agelaki and colleagues, opening to the possibility of monitoring the changes on CTCs during treatment with targeted agents [70]. Of note, in the multicenter phase II clinical trial from Pestrin et al. patients were defined as having HER2-positive CTCs if 50% of CTCs had HER2 overexpression and/or amplification [35].

Similar negative results have been observed with the use of the ADC trastuzumab-emtansine (T-DM1) in the phase II CirCe T-DM1 study [71]. In this trial, CTCs were considered HER2-positive based on the presence of ≥1 *ERBB2* amplified CTCs. Among the 155 heavily pretreated HER2-negative MBC patients screened, 9% had HER2-positive CTCs and 11 received T-DM1. Only one patient achieved partial response, four patients had stable disease as best response, while the median PFS (mPFS) and OS were 4.8 and 9.5 months, respectively. Another single-arm trial evaluated the administration of anti-HER2 therapy in HER2-negative MBC with HER2-positive CTCs defined by FISH [36]. Sixty-nine of 311 patients (22%) had HER2-positive CTCs and twenty received trastuzumab in combination with vinorelbine. The clinical activity of this anti-HER2 regimen was low, with a mPFS of 2.7 months, and an objective response rate (ORR) of 5%, considerably lower as compared to the 44–86% ORR reported with this combination regimen in HER2-positive MBC [72]. Of note, the CTC isolation method used in this study led to consider both CK-positive and CK-negative CTCs and is not known whether the latter may act differently to classic HER2-amplified BC cells in terms of response to anti-HER2 treatment.

#### HER2 expression on CTCs and therapy resistance in MBC

While the presence of HER2-positive CTCs highlights the intra- and inter-tumoral HER2 expression heterogeneity, their specific influence on treatment response in HER2-negative MBC is still not fully understood. Low HER2 expression in HR-positive MBC has been associated with a diminished response to endocrine therapy (ET). A retrospective study demonstrated a shorter mPFS in patients with HR-positive/HER2-low when compared to HR-positive/HER2-negative MBC receiving the combination of ET and a CDK4/6 inhibitor (CDK4/6i) (8.9 versus 18.8 months, respectively) [73]. Similarly, an intrinsic subtype molecular analysis of the MONALEESA phase III clinical trials, demonstrated that the HER2-enriched subtype was associated with a 2.3-fold increased risk of disease progression from therapy with the CDK4/6i ribociclib when compared to luminal A subtype (*i.e.*, HR-positive/HER2-negative) [74]. These observations raise the possibility of using HER2 expression as a candidate biomarker to predict resistance to ET in HR-positive/HER2-negative MBC. In this instance, CTCs have emerged as non-invasive candidates for studying tumoral HER2 expression and its dynamic changes over time. Supporting this notion, Roβwag et al. conducted a study where they isolated CTCs from a patient with HR-positive/HER2-negative MBC that had experienced disease progression while on ET [75]. The researchers confirmed that the isolated CTCs were refractory to ET *in vitro* and observed that inhibiting estrogen receptor signaling resulted in an upregulation of HER2 expression in the CTCs. They also showed that HER2 expression in CTCs is relevant for cell growth and survival. Nevertheless, pharmacological inhibition of HER2 with lapatinib only modestly reduced cell proliferation which may suggest that acquired overexpression of HER2 coexists with adaptive mechanisms that enable these CTCs to escape HER2 inhibition. Similarly, Jordan et al. documented the presence of HER2-positive CTCs in 84% (n = 16/19) of patients with HR-positive/HER2-negative MBC after multiple lines of therapy [76]. The proportion of HER2-positive CTCs relative to HER2-negative CTCs increased as the disease progressed. Cultured cell lines were established using CTCs, resulting in distinct populations of HER2-positive and HER2-negative CTCs. The HER2-positive CTCs exhibited a higher proliferation rate, as indicated by the expression of the proliferation marker Ki-67. Interestingly, HER2-positive CTCs did not demonstrate increased sensitivity to the HER2 inhibitor lapatinib compared to HER2-negative CTCs, suggesting a reduced dependence on HER2 signaling, unlike commercially available BC cell lines with HER2 amplification. Furthermore, tumors generated from purified HER2-positive CTCs were larger and exhibited a higher frequency of visceral metastases in mice compared to tumors generated from HER2-negative CTCs. Overall, these findings shed light on the potential role of HER2 expression in CTCs in predicting therapy resistance in HR-positive/HER2-negative MBC. Additionally, the characteristics of these emerged HER2-positive CTCs provided insights into the *in vitro* response to HER2 inhibitors. Further research is needed to fully understand the clinical implications and therapeutic implications of HER2-expressing CTCs in this patient population.

## Conclusions and future perspectives

Given the central role of HER2 in breast oncology it is essential to accurately evaluate the HER2 status of the tumor before any treatment planning by developing sensitive and reliable methods for HER2 testing and scoring. HER2 expression can be determined in a non-invasive manner through CTCs, detected in the peripheral blood of BC patients, as a “*real-time* biopsy” [77]. CTCs have the potential to allow repeated sampling and avoid confounding and biologically relevant effects such tumor heterogeneity of tissue biopsy. Nonetheless, along with opportunities presented by CTCs, new pitfalls must be addressed.

One of the main limitations of the body of literature available to date is the lack of a univocal definition of HER2-positive CTCs. Several studies found that HER2-positive CTCs are detected in a subset of patients with HER2-negative BC, however, the functional significance of these cells remains uncertain and the number of CTCs that could justify the use of anti-HER2 is still an open question. It is possible that a stricter definition of HER2-positive CTCs would identify a group of patients with HER2-negative BC who would benefit from anti-HER2 treatment. Moreover, the landscape of anti-HER2 therapies has evolved significantly in the last years [5] and targeting HER2-positive CTCs with novel drugs such as T-DXd may allow to reach clinically meaningful results. Also, the reported activity of T-DXd in patients with HER2-zero MBC [78] might change the way we look at HER2 also on CTCs since a minimal membrane expression could be sufficient to enable the targeted delivery of the cytotoxic payload of ADCs.

Beyond response to anti-HER2 targeted agents, exploring HER2 status on CTCs might lead to a deeper comprehension of the mechanisms of resistance to CDK4/6i combinations both, in advanced and adjuvant settings, that represent a major hindrance in the treatment of HR-positive/HER2-negative BC patients.

Moreover, once established a method to define HER2 expression on CTCs different biomarkers could be tested on CTCs to guide personalized treatment of BC patients. For instance, an exploratory biomarker analysis from the phase II ICARUS-Breast01 trial revealed that a higher baseline count of HER3-positive CTCs, as well as their reduction during treatment with Patritumab Deruxtecan (HER3-DXd) was associated with increased likelihood of achieving an early response [79].

The main limitation of CTCs as a single biomarker is the limited number of cells detected in the peripheral blood, especially in the early setting. Therefore, a comprehensive liquid biopsy approach using, along with CTCs, other circulating tumor components including circulating tumor DNA [[80], [81], [82], [83], [84]], and tumor-extracellular vesicles [85] among others, may increase the sensitivity and specificity of determining HER2 status and its clinical implications. An advantage of CTCs over other liquid biopsy analytes is the possibility to perform DNA, RNA, and protein analyses on a single cell level [86,87], thus enabling evaluation of intrapatient heterogeneity of HER2 expression and providing insight into biological processes.

## Authors’ disclosures of potential conflicts of interest

**GC** served as consultant or advisor for Roche, Lilly and Bristol-Myers Squibb, served on the speaker's bureau for Roche, Pfizer and Lilly, received travel funding from Pfizer and Roche and received honoraria from Roche, Pfizer, Lilly, Novartis and Seagen, all outside the submitted work. **MC** reports personal fees from Lilly, Sermonix, Data Genomics, Foundation Medicine, Guardant Health, Celcuity, Iylon, and Ellipses and grants and personal fees from Pfizer, AZ and Menarini, all outside the submitted work. No other potential conflicts of interest are reported.
